# Assessment of the Effect of PLGA Co-polymers and PEG on the Formation and Characteristics of PLGA-PEG-PLGA Co-block Polymer Using Statistical Approach

**DOI:** 10.15171/apb.2019.045

**Published:** 2019-08-01

**Authors:** Teuku Nanda Saifullah Sulaiman, Dwi Larasati, Akhmad Kharis Nugroho, Syaiful Choiri

**Affiliations:** ^1^Department of Pharmaceutics, Universitas Gadjah Mada, Sekip Utara, Yogyakarta, Indonesia 55281.; ^2^Faculty of Pharmacy, Universitas Gadjah Mada, Sekip Utara, Yogyakarta, Indonesia 55281.; ^3^Pharmaceutical Technology and Drug Delivery, Department of Pharmacy, Universitas Sebelas Maret, Ir. Sutami 36A, Surakarta, Indonesia, 57126.

**Keywords:** PLGA-PEG-PLGA, Co-block polymer, Nano polymeric micellar, Thermo- sensitive polymer

## Abstract

***Purpose:*** To assess the effect of the lactic acid (LA)-to-glycolic acid (GA) molar ratio and polyethylene glycol (PEG) concentration on the formation of poly-lactide co-glycolide acid (PLGA)-PEG-PLGA co-block polymers simultaneously using statistical approach.

***Methods:*** A 2^2^ full factorial design with the addition of a point in the center of the design, namely curvature, was applied. Fourier transform infrared (FTIR), differential scanning calorimetry (DSC), and nuclear magnetic resonance (NMR) were performed to confirm the formation of the co-block polymer. Simvastatin (SMV), a drug model was incorporated into the nano-polymeric micellar (NpM) of PLGA-PEG-PLGA followed by solubility phase, particle size, zeta potential, and entrapment efficiency characterizations.

***Results:*** FTIR, DSC, and NMR successfully confirmed the formation of co-block polymers. Solubility of SMV increased from 2 to 44-folds depending on co-block concentration with entrapment efficiency of 59%-80%. The NpM had size in the range of 206 to 402 nm with negative zeta potential. LA to GA ratio had greater effect on particle size reduction and increasing of co-polymer length. In addition, it had higher contributions on increasing of solubility and entrapment efficiency of SMV than PEG.

***Conclusion:*** According to these findings, the LA to GA ratio and PEG concentration gained a great consideration in order to prepare the PLGA-PEG-PLGA co-block which fulfilled the quality target product profile of NpM delivery system.

## Introduction

Recently, delivering a guest molecule to a target site is either attractive owing to major challenges and advantages or a new perspective. Several hurdles related to either guest molecule characteristics or physiological condition have emerged.^1,2^ Drugs have several issues related to physicochemical properties, by which they are affected with respect to the design of the delivery system. The purpose of the delivery system is to enhance the bioavailability of a drug by enhancing solubility and absorption, controlling drug release, or protecting them against stability issues, e.g., gastrointestinal environment and ambient conditions.^3^ For instance, bioavailability is the main problem and one factor promoting failure in the drug development process. Low bioavailability is mainly influenced by poor water solubility followed by low permeability issues, e.g., high hydrophilicity, poly glycoprotein (p-gp) efflux, and transport-mediated issues.^4-7^

Furthermore, as a consequence of the nano-polymeric micellar (NpM), a polymer core nanoparticle has been introduced to overcome these limitations. An NpM consists of an amphiphilic compound aggregate where it contains a lipophilic compartment in the inner part (core aggregate) and a hydrophilic compartment in the outer part of the polymer structure.^8-10^ Like a surfactant, it can enhance bioavailability by elevating solubility and enhance permeation by hindering p-gp efflux owing to modifications of the drug surface by encapsulating with a polymer and steric protection.^11,12^ It can be modified to increase the lipophilicity of lipophilic groups to alter the transport mechanism.^9,13^

In an NpM, co-block polymer plays a fundamental rule in determining the successful delivery and characteristics of the final product.^14^ In order to achieve an optimal delivery system, modification of the polymer is carried out to obtain an appropriate feature.^15-17^ One of them, a lipophilic and biodegradable polymer, polylactide-co-glycolide acid (PLGA) has been combined and reacted with polyethylene glycol (PEG) to achieve a co-block polymer, in which it has hydrophilic and lipophilic sides. Co-block polymer of PLGA-PEG-PLGA has been reported by several researchers as a carrier for a targeted delivery system.^15,18^ It had been successfully applied via the injectable formulation,^19^ ocular delivery,^20^ peptide delivery,^21^ and lung delivery.^22^ In addition, this polymer can be applied for long-term and sustained-release effects as well as via localized chemotherapeutic agents to reduce toxicity.^23^ However, on the market, there is wide variation based on molecular weight and constituent ratio of PLGA. This polymer has been reported to have a sol-gel transition owing to the temperature effect, thus it is called a thermo-sensitive polymer. This ability is a fundamental trait for enhancing the controllability of drug release, which the viscosity increases owing to a sol-to-gel transition at body temperature.^15,19,21,24^

The PLGA-PEG-PLGA co-block polymer is prepared by an opening ring of the constituent components of PLGA and polymerization followed by a self-arrangement of PLGA and PEG to obtain a tri-block polymer.^25,26^ Additionally, the ratio of constituent components of PLGA, namely _D-L_*-*lactide (LA) and glycolide (GA), features prominently and determines the characteristics of PLGA.^19,26^ The amount of monomer in one block of the polymer is affected by PLGA formation and the type and concentration of PEG. To date, the preparation method and synthesis of PLGA-PEG-PLGA have been described extensively. Nevertheless, there was no reported study assessing either factor or its interaction with PLGA-PEG-PLGA co-block formation simultaneously using a design of experiment (DoE) approach. In this study, we assessed both factors using DoE for simultaneous evaluation and established the optimized condition that fulfills the quality target product profile of NpM under wide range of the LA-to-GA ratios and PEG concentrations. Thereafter, the main effect and interaction of both factors can be obtained. Hence, the purpose of this study was to assess the co-block polymer behavior and characteristics of an NpM, which was affected by PLGA formation (constituent components ratio) and PEG concentration through a factorial design approach.

## Material and Methods

### Materials

LA and GA were purchased from Tokyo Chemical Industry (Tokyo, Japan). PEG 1000, methanol, and dichloromethane were purchased from Merck (Darmstadt, Germany) and staneous-2-ethyl hexanoate (catalyst) was purchased from Sigma Aldrich (St. Louis, MO). Simvastatin (SMV) was obtained from Dexa Medica (Palembang, Indonesia) as a gift simple.

### Experimental design for preparing PLGA-PEG-PLGA co-block polymers

A 2^2^ -factorial design (FD) comprising four design points was applied to assess and elucidate the main effect and interaction of the LA-to-GA ratio (molar ratio) and PEG concentration at two levels. It was applied to determine on the formation and characteristics of micellar PLGA-PEG-PLGA, e.g., molecular vibration, thermal behavior, solubility enhancement, particle size and its distribution, and entrapment efficiency. Factors and their levels of the design are presented in Table 1. In order to enhance the predictive power of the model and determine the requirement of the additional level, a design point was added to the middle of the design as a curvature. The main effects and interactions were calculated according to multiple linear regression analysis (MLRA). A contour plot was constructed depending on the model equation. The gradual color (from high value; red, to low value; blue) of the contour plot depicted the predicted value of each response based on its equation.

**Table 1:** Experimental design of 2^2^ full factorial design with curvature for preparation of nano polymeric micelle delivery system

**Types**	**Formulation code**	**LA to GA molar ratio**	**PEG 1000 (%) (w/w)**
Design points	F1	2	30
	F2	10	30
	F3	2	60
	F4	10	60
Additional point	Curvature (F5)	6	45

### Preparation of PLGA-PEG-PLGA co-block polymers

PLGA-PEG-PLGA was prepared according to Zentner’s method with a minor modification.^25^ Briefly, PEG 1000 was placed into a filter flask and vacuumed under a stirring condition at 130°C for 5 hours. Thereafter, LA and GA were added to the melted PEG until all components melted and a homogeneous mixture was achieved, then stannous 2-ethyl hexanoate was added to the mixture as a catalyst and stirring was carried out for 5 hours under similar conditions. Furthermore, the purification step was conducted depending on the thermo-sensitive characteristics of PLGA-PEG-PLGA. The polymer was added to cold water (4°C) followed by filtration using a 0.45 µm membrane filter. The filtrate was heated until 70°C followed by centrifugation at 10000 x g for 15 minutes and the sediment was collected. The purification step was repeated three times. Finally, the rest water was eliminated through a Thermo PowerDry LL1500 lyophilizer (Waltham, MA, USA) at a pressure of 0.01 mBar and temperature of -50°C. Yield was calculated according to the theoretical polymer weight.

### Characterization of PLGA-PEG-PLGA co-block polymers

The vibrational spectrum of PLGA-PEG-PLGA co-block polymers was analyzed with a Thermo Nicolet i50 Fourier transform infrared (FTIR) spectrophotometer (Waltham, MA, USA). The FTIR was equipped with attenuated total reflectance (ATR) with ZnSe crystals and deuterated triglycine sulphate detectors. The sample was placed on the ATR crystal and scanned from 650–4000 cm^-1^ with a resolution of 2 cm^-1^ and 32 iterations as a duplicate.

The thermal behavior of the LA, GA, PEG, and PLGA-PEG-PLGA co-block polymers were characterized with a Shimadzu DSC-60 differential scanning calorimetry (DSC) and Shimadzu DTG-60 thermal gravimetric analyzer (Kyoto, Japan). Approximately 10 mg of sample (PLGA-PEG-PLGA co-block polymer without adding any water) was placed into an Al_2_O_3_ seal pan and heated from 0 to 200°C at a rate of 10°C/min under a 30 mL/min nitrogen atmosphere. An empty pan was used as a reference.

### Preparation of simvastatin nano-polymeric micellar

A poorly water-soluble drug, SMV, as a drug model, was incorporated into the NpM using an emulsification method followed by a solvent evaporation technique. Firstly, a 1 mg SMV was dissolved in dichloromethane and added to the 0.05% PLGA-PEG-PLGA solution under a stirring condition at 1000 rpm for 30 minutes. In order to eliminate the DCM, the stirring condition was at ambient temperature overnight.

### Characterization of simvastatin loaded polymeric micelle

The NpM was characterized by solubility profile, particle size, and distribution, zeta potential, and entrapment efficiency. An excess amount of SMV (10 mg) was added to 10 mL of 0.005, 0.01, 0.05, 0.1, 0.5 and 1% PLGA-PEG-PLGA co-block solution. The mixture was stirred at ambient temperature (26±2°C) for 48 hours followed by centrifugation at 10 000 x g for 30 min. The supernatant was analyzed spectrophotometrically under a validated analytical method. The solubility profile was constructed according to the co-block polymer concentration and saturated solubility of SMV.

Particle size and distribution along with zeta potential were measured using a Malvern Nano ZS particle size analyzer (Malvern, UK) with a dynamic light-scattering technique at a wavelength of 632 nm, scattering angle of 173°, and refractive index of 1.333. Zeta potential was calculated using a similar instrument depending on electrophoretic mobility.

Entrapment efficiency (EE) was assessed according to the thermo-sensitive behavior of the polymer during the heating process. The polymeric micelle was heated at 37±1°C for 30 minutes followed by centrifugation at 10 000 × g for 15 minutes. A 3 mL amount of supernatant was withdrawn and analyzed spectrophotometrically under a validated analytical method. EE (%) was calculated according to the percentage of drug in the micelle versus the theoretical drug ratio. Meanwhile, the drug load (%) was calculated according to the percentage of the drug in the micelle relative to the amount of total micelle.^27^ All characterizations were performed triplicates.

### Optimization and NMR characterization

The two-factor interaction model comprised the main effects and the interactions were generated for all responses to the MLRA approach. Each model was evaluated based on the goodness-of-fit parameters, including the determination coefficient (R^2^), adjusted determination coefficient (Adj. R^2^), predicted determination coefficient (Pred. R^2^), adequate precision (adeq. prec.) and predicted residual error sum square (PRESS).

A significant effect on response was determined by F-test or *P* value of analysis of variance (ANOVA), which was calculated via Design-Expert software (Stat-Ease Inc., Minneapolis, MN) with a confidence level of 95% (*P* = 0.05). A contour plot or 3D-contour plot was constructed based on the model of each response. A superimposed contour plot was employed to determine the optimized region based on product performance by combining the contour plot of each model. Verification was performed statistically by comparing the predicted results and observed results using a one-sample t-test with a confidence level of 95% (*P* = 0.05).

The optimized PLGA-PEG-PLGA was characterized by proton (^1^H) and carbon (^13^C) nuclear magnetic resonance spectroscopy (^1^H-NMR and ^13^C-NMR) for structural confirmation. A solution state of ^1^H-NMR and^13^C-NMR using a JEOL EC ZR 500 MHz spectrometer (Tokyo, Japan) was performed. The optimized PLGA-PEG-PLGA was dissolved in deuterated dimethyl sulfoxide (DMSO-D_6_) until a concentration of 100 mg/mL was achieved. The sample was set at a temperature of 25°C and an iteration of 10 000 scans. Baseline correction with a polynomial model, integration, and chemical shift correction was carried out via Mnova 14 software (Mestrelab Research; Santiago de Compostela, Spain).

## Results and Discussion

### Preparation of PLGA-PEG-PLGA co-block polymer

**Scheme 1:**
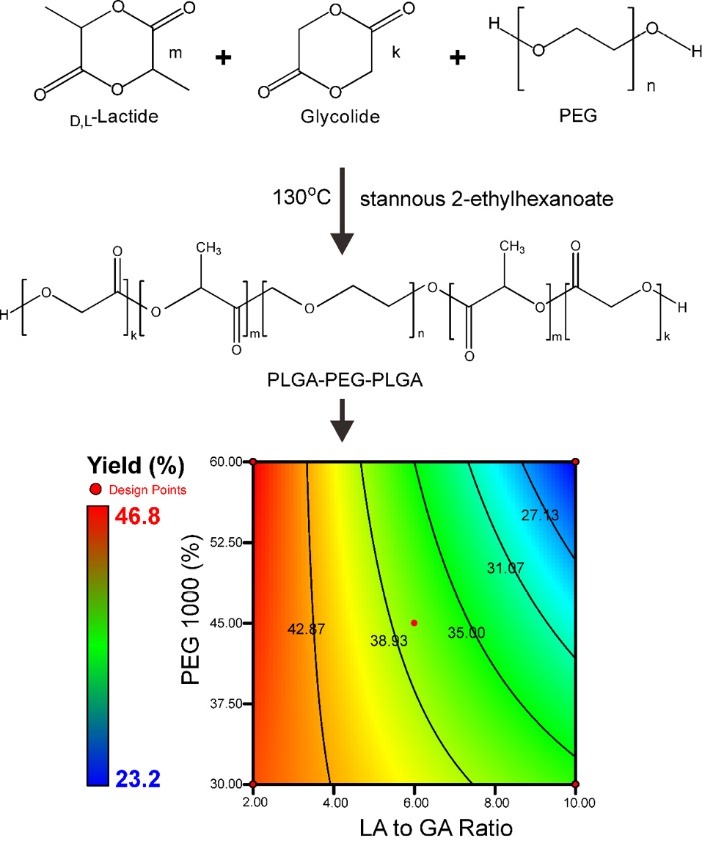
Formation of PLGA-PEG-PLGA co-block polymer.

Preparation of PLGA-PEG-PLGA co-block polymer proceeded according to Scheme 1. The reaction involves an opening of the ring of the LA and GA and formation of the PLGA co-polymer followed by self-assembly to form the co-block of PLGA-PEG-PLGA. All co-blocks were similar in terms of their consistencies, i.e., semi-solid and their color, i.e., clear yellow, but it differed from their yields. A similar result as a confirmation of the organoleptic characteristics of PLGA-PEG-PLGA co-block polymer was reported.^25^ The yield of PLGA-PEG-PLGA is also presented in Scheme 1. The contour plot depicts the effect of the LA-to-GA ratio and PEG concentration on the yield. The highest yield was obtained at LA-to-GA ratio of 2:1 with PEG concentration in the range of 30-60%. Meanwhile, the lowest yield was obtained at LA-to-GA ratio of 10:1 and PEG concentration of 60%. This indicated that at a low LA-to-GA ratio, PEG did not significantly influence the yield (*P *> 0.05). Moreover, at a high level of LA-to-GA ratio and PEG, it was determined that the yield was altered as a consequence of its concentration. High yield in the preparation of PLGA-PEG-PLGA co-block polymer is affected by thermo-sensitive characteristic.^28,29^ Failure of formation of PLGA-PEG-PLGA reduced the yield based on eliminating non-thermo-sensitive material.^19,26^ In addition, formation of PLGA-PEG-PLGA depended on the concentration of GA in the system. Therefore, an increase in LA diminished the yield because of excess unreacted material.

### FTIR characterization

**Figure 1:**
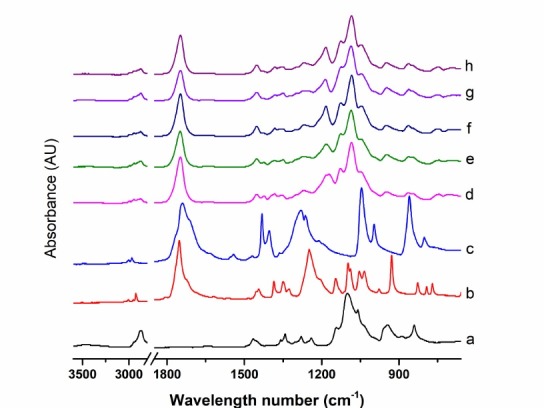
ATR-FTIR spectra of PEG 1000 (a), _D-L_-lactide (b), glycolide (c), F1 (d), F2 (e), F3 (f), F4 (g), and F5 (h).

The FTIR spectra of PEG, LA, GA and co-block polymer are presented in Figure 1. The specific vibrational peaks of PEG were assigned at 1465.0 and 3462.9 cm^-1^ according to C-H bending and O-H stretching vibrations, respectively. Meanwhile, the specific vibrational peaks of LA and GA were assigned at wavenumbers of 1752.4 and 1740.1 cm^-1^, corresponding to the C=O ester vibration, 1097.5 and 1045.4 cm^-1^corresponding to the C-O vibrations, along with 1444.1 and 1431.5 cm^-1^ corresponding to the alkyl bending vibrations for LA and GA, respectively. In addition, all co-block polymers had specific vibrations for PEG, LA, and GA, but shifted to different wavelength numbers, i.e., C=O ester vibration ranging from 1749.1 to 1747.7 cm^-1^, C-O vibration ranging from 1086.1 to 1083.8 cm^-1^, alkyl bending vibration of approximately 1542 cm^-1^ as well as the O-H stretching vibration at 3468 cm^-1^ corresponding to the end group of PLGA co-polymers. There was no significant shifting of the specific vibrational peaks of the five co-block formulations. A similar result was reported and confirmed with these results surrounding the formation of PLGA-PEG-PLGA.^18^ The specific functional groups of PLGA co-block polymers through ATR-FTIR result appeared and demonstrated the structural confirmation of co-block polymers.

### Differential scanning calorimetry characterization

**Figure 2:**
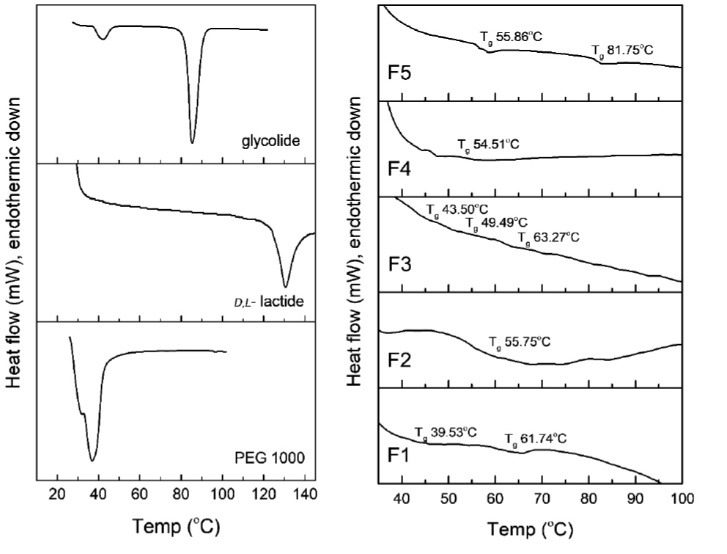
DSC thermogram of PEG 1000, *D,L*-lactide, glycolide and PLGA-PEG-PLGA co-block polymers (F1-F5).

The DSC thermogram of LA, GA, PEG, and co-block polymers is presented in Figure 2. Sharp endothermic peaks assigned as the melting points of PEG, LA, and GA were observed at 36.93, 128.5, and 85.34°C, respectively. All specific endothermic peaks were not found in the co-block thermograms. The sharp endothermic peak transformed to a weak endothermic phenomenon, known as the glass transition temperature (T_g_). The higher T_g_, the longer the co-block length was.^30^ The T_g_s of the F1 co-block were found at 39.53 and 61.74°C, meanwhile F2 only had one T_g_ at 55.75°C. In addition, three T_g_s were found in the F3 thermogram, i.e., 43.50, 49.49, and 63.27°C. F4 had a single T_g_ at 54.51°C and F5 had two T_g_s at 55.86 and 81.75°C.

In order to elucidate the effect of LA-to-GA ratio and PEG concentration, the contour plot of T_g_ which had the highest enthalpy (the main component in the polymer length mixture) for each co-block was constructed (data not shown). The lowest T_g_ was obtained at a low level of LA-to-GA ratio (2:1) and PEG concentration (10%), while the highest T_g_ was observed at a high level of LA-to-GA ratio (10:1) and PEG concentration of 30%-60%. At the low LA-to-GA ratio, the PEG concentration determined on the T_g_ of the co-block polymer. Notwithstanding, PEG did not have a significant effect on the T_g_ of the co-block at high LA-to-GA ratios. The increasing of the LA-to-GA ratio elevated T_g_ owing to rising hydrophobic interactions and molecular weight of the polymers. In addition, this result was bolstered by a previous reported study.^31^ PEG contributed to reducing T_g_ owing to crystallinity alteration.^27^ Constituent components had a great impact on the formation of PLGA-PEG-PLGA. Those factors affected the formation of single or more systems of polymer lengths according to the stoichiometric reaction, sequence formation of PLGA and temperature/duration of synthesis of the co-block polymers.^32^

### Statistical fitting parameter of DoE

Previous studies reported that DoE was very useful for optimizing a formulae/condition and assessing the studied factor with regards to responses.^33,34^ In order to apply DoE for optimizing and assessing the LA-to-GA molar ratio and PEG concentration on quality target product profiles of NpM, the model should be fit well. The best fitting model should meet several requirements: (1) the model was statistically significant (*P *< 0.05); (2) R^2^ was more than 0.7; (3) the difference between Adj. R^2^ and Pred R^2^ was not more than 0.2; and (4) adequate precision should be at more than 4.^33^ Additionally, the curvature as an additional design should be not significant (*P *> 0.05) for a linear function. However, further discussion and evaluation could be performed for significant curvature. All statistical parameters are summarized in Table 2. All parameters met the requirements of the goodness of fit (Point 1-4). However, the zeta potential model did not vary significantly owing to a narrow range of results. The significant curvature indicates that the model had a non-linear function which requires three or more levels to evaluate.

**Table 2:** Statistical parameter of factorial design for nano-polymeric micelle delivery system

**Parameters**	**Solubility (µg/mL)**		**Particle size (nm)**		**PDI**		**Entrapment efficiency (%)**	
	**Coef.**	***P *** **value**	**Coef.**	***P *** **value**	**Coef.**	***P *** **value**	**Coef.**	***P *** **value**
Intercept	19.76	-	278.25	-	0.52	-	69.01	-
A	1.09	0.0221	-56.13	<0.001	-0.24	<0.001	2.64	0.002
B	-12.79	<0.001	-35.83	<0.001	0.13	<0.001	-6.99	<0.001
AB	-2.78	<0.001	25.38	<0.001	-3.4x10^-3^*	0.794	0.14	<0.001
Model		<0.001		<0.001		<0.001		<0.001
Curvature		0.520*		<0.001		<0.001		0.769*
R^2^	0.9907		0.9956		0.9791		0.9623	
Adj. R^2^	0.9880		0.9942		0.9728		0.9509	
Pred. R^2^	0.9792		0.9900		0.9530		0.9151	
Adeq. Prec	38.77		61.07		29.23		20.58	

A = _D-L-_lactide to glycolide ratio; B = PEG 1000; PDI = polydispersity index. Coef. = regression coefficient; R^2^ = coefficient of determination, Adj. R^2^ = Adjusted R^2^; Pred. R^2^ = Predicted R^2^; Adeq. Prec. = adequate precision. * Statistically not significant difference (*P *> 0.05).

### The effect on solubility behavior

**Figure 3:**
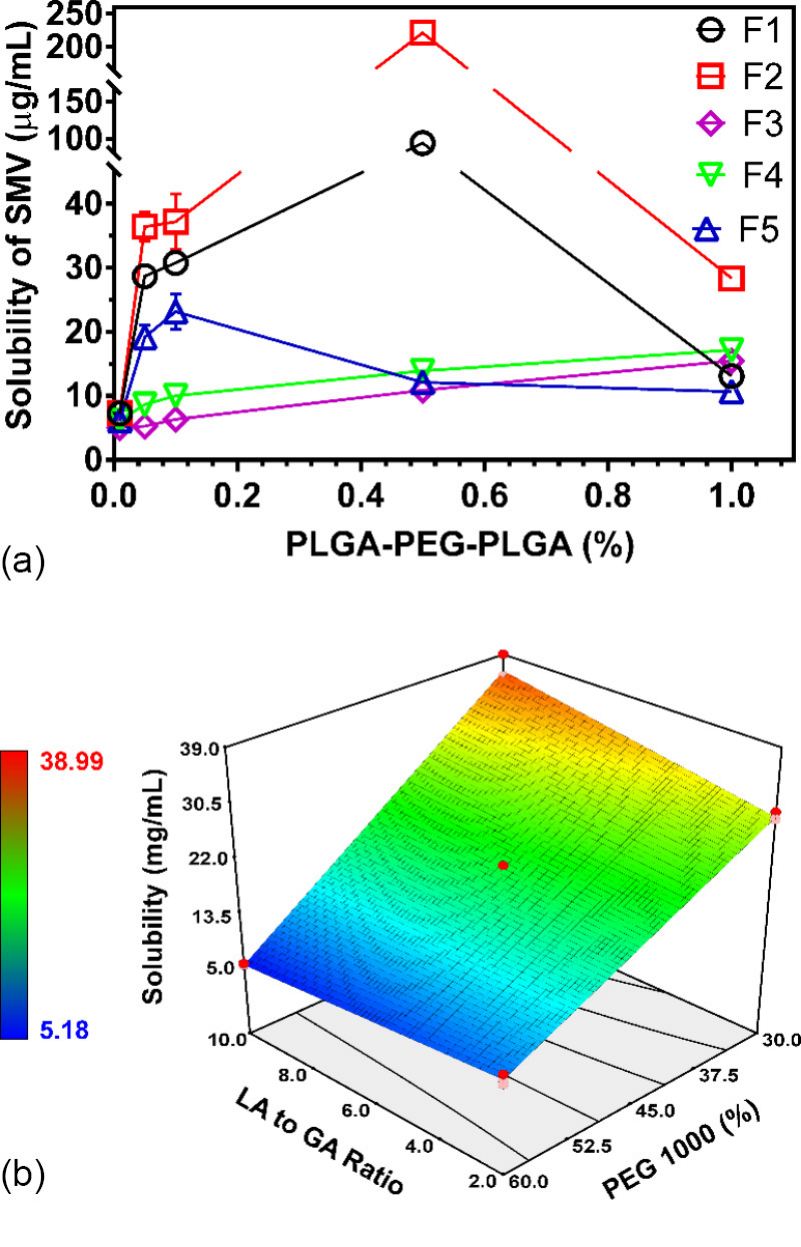
Solubility profile of simvastatin under different concentration of PLGA-PEG-PLGA co-block polymers (a) and contour plot of solubility at 0.05% (b).

SMV had low solubility in water (5.04±0.30 µg/mL). It can be enhanced and encapsulated using the NpMDS. Interaction between SMV and lipophilic moiety in PLGA-PEG-PLGA co-block polymer through encapsulation mechanism promoted an increase in the solubility of SMV. The solubility profile of SMV at different polymer concentrations is presented in Figure 3a. F2 had the highest solubility enhancement among these co-block polymers, a roughly 44-fold increment at 0.5% (w/v) of polymer concentration. The critical micelle concentration (CMC) of PLGA-PEG co-block polymer was reported by Zhu et al. at approximately 0.0071% (w/v).^27^ In another report, the CMC of PLGA-PEG-PLGA was 0.01-0.03% (w/v).^32^ In this study, the effect of the concentration above CMC (0.01% w/v) increased solubility dramatically (Figure 3a). The high solubility profile was observed at low levels of PEG concentration. Low to medium levels of PEG had a quadratic shape of solubility profiles. Although linear function increasing the polymer concentration was observed at a high level of PEG concentration, it had a low enhancement of SMV solubility at roughly two to four folds owing to aggregation of micelles. The solubility behavior was affected by the formation of a PLGA structure in the co-block, which was affected by LA-to-GA ratio more so than hydrophilic groups.

In order to elucidate the effect of the LA-and-GA ratio and PEG concentration on solubility behavior, the solubility of SMV at a 0.05% polymer concentration was employed to construct the appropriate model depending on the MLRA. The solubility model met the requirement; thus it could be used to predict and assess those factors. All statistical parameters of the solubility model are listed in Table 2. The solubility of SMV in 0.05% of polymer solution ranged from 5.2 to 39.0 µg/mL. The LA-to-GA ratio (+1.09) had a significant effect on increasing solubility (*P* < 0.05), while the PEG concentration (-12.79) had a negative effect on solubility of SMV (*P* < 0.05). The significant interaction between both factors was observed by reducing solubility of SMV (*P* < 0.05). In order to elucidate the interaction, a 3D-contour plot was constructed and is presented in Figure 3b. The lowest solubility was observed at high levels of PEG and all LA-to-GA ratios. Diminishing PEG concentration increased solubility of SMV. Therefore, the highest solubility was obtained at the highest LA-to-GA ratio and lowest PEG concentrations. This showed that the influence of PEG was higher than that of the LA-to-GA ratio on solubility. PLGA-PEG-PLGA had two sides comprising hydrophilic groups, PEG, in the middle block and lipophilic groups, with PLGA at the sides of the co-block. The length, block type, branching, and cross-linking of co-block polymer governed the hydrophilicity and lipophilicity, thus influencing micellar formation during hydration.^14,16,26^ The hydration state of co-block polymer, a micellar structure, could entrap a lipophilic moiety into lipophilic groups. Therefore, it could improve the solubility of SMV. SMV was entrapped within lipophilic groups of co-blocks, thus it depended on hydrophilic and lipophilic characteristics of co-blocks.^17,22^

### The effect on particle size and distribution

**Figure 4:**
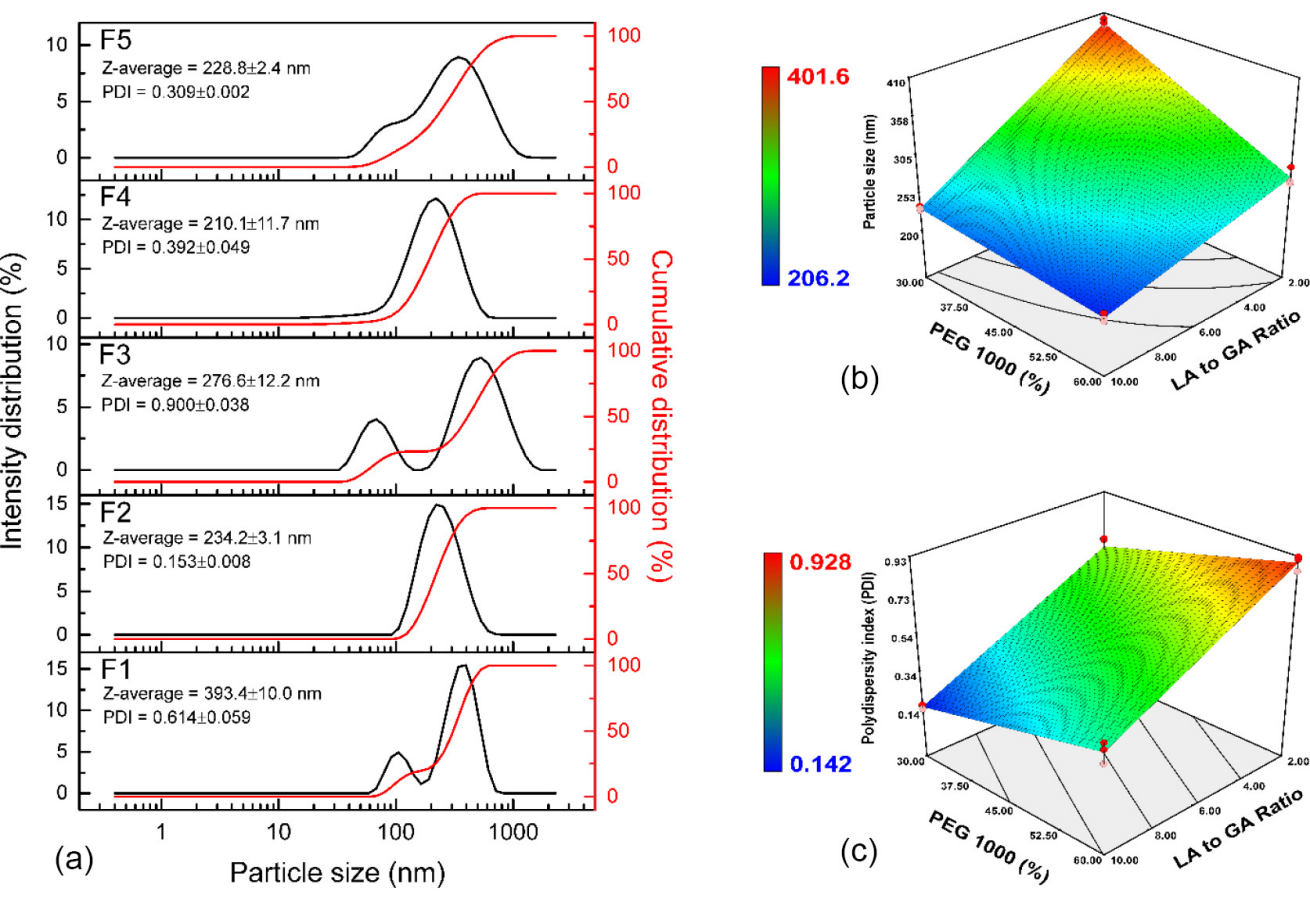
Particle size distribution of NpMDS (F1-F5) (a) and contour plots of particle size (b), and polydispersity index (c).

Formation of micelles during dilution was fundamentally affected by co-block formation. The bending of co-block and their aggregation to form a micelle had a great impact on particle size and distribution. Directly, it was caused by the length of the co-block and its position.^32,35^ The particle size distribution of co-block polymer is portrayed in Figure 4a. The particle size distribution was shown to have heterogeneous characteristics depending on the level of each factor. The low LA-to-GA (F1 and F3) ratio promoted the polydisperse system, where the two peaks of particle size distribution were observed. Meanwhile, a monodisperse system was observed at a high LA-to-GA ratio (F2 and F4). In addition, the middle level of the design (F5) had a combination of both characteristics. Moreover, it exhibited a polydisperse system without a robust separation of two peaks. The cumulative particle size distribution suggested the distribution of particle size. The narrowest distribution was observed at F2 followed by F4. The broad distribution was observed at F3 and F1 with two sigmoidal phases of distribution. Meanwhile, F5 had a broad distribution with one sigmoidal phase of cumulative particle size distribution. The distribution of particle size was mainly caused by variation of co-block length. During hydration, each co-block assembled spontaneously to form micellar structure. Longer co-block length had greater particle size than shorter co-block length. However, some micellar structure consisted of the co-block with different length in order to stabilize the micellar structure thermodynamically.^32^ Nevertheless, over proportion in the co-block length variation promoted broadening of particle size distribution.

The particle size of the NpM ranged from 206.2 to 401.6 nm. According to the MLRA of particle size, the model met the aforementioned requirements. However, the curvature was significant (*P* < 0.05). All factors had a positive impact on decreasing the particle size (*P* < 0.05). The effect of LA-to-GA ratio (-56.13) on particle size was higher than that of PEG concentration (-35.83). The interaction of both factors increased the particle size of NpM (*P* < 0.05). The contour plot of particle size is found in Figure 4b. The region to produce the highest particle size was found at low PEG and LA-to-GA ratio. Meanwhile, the smallest particle size was obtained at high LA-to-GA ratios and PEG concentration. According to thermal analyses, this proved that there was no single polymer length system in any co-block, hence aggregation formation varied depending on the length of the co-block polymer. Not only particle size, but also distribution of particle size was impacted by variation of polymer length.^26^

Distribution of particle size is depicted by polydispersity index (PDI). PDI of all co-blocks ranged from 0.142-0.928. According to the MLRA approach of PDI, the model met the aforementioned requirements. Yet, the curvature was found to be significant (*P* < 0.05). The significance of curvature in terms of particle size and distribution suggested that there was no linear function, therefore a quadratic function should be implemented.^34^ Hence, particle size and distribution in the middle of the design did not follow the model. In addition, the verification of the model should be not significant owing to the significant curvature. Although, a high residual between observed and predicted was accomplished at high values of particle size and PDI while low residual was achieved at low values of particle size and PDI.

The LA-to-GA ratio (-0.24) had a statistically positive effect on reducing PDI (*P* < 0.05). Meanwhile, the PEG concentration (+0.13) had a negative effect on increasing PDI statistically (*P* < 0.05). In addition, the interaction of both factors had no significant impact on increasing PDI (*P* > 0.05). The 3D-contour plot of PDI is presented in Figure 4c. The monodisperse system was found at high LA-to-GA ratio and low PEG concentration. On the other hand, higher PDI correlating to the polydisperse system was obtained at a low LA-to-GA ratio and PEG concentration. Moreover, PDI was not significantly affected by the LA-to-GA ratio at mid to high level of PEG concentration. The distribution of aggregate of NpM was affected by co-block polymer length,^27^ which was confirmed by DSC analysis in that it was a similar pattern to the amount and value of T_g_ for each co-block.

### The effect on surface charge of nano-polymeric micelle

The surface charge of the NpM contributed to physical stability and easy transport mechanisms. The surface charge of NpMDS is indicated by the zeta potential. The zeta potential of all co-blocks ranged from -21.9 to -14.5 mV. The negative charge of the particle surface was affected at the end group of the PLGA co-polymer. A similar result was reported such that negative charge (-24 until -15 mV) of PLGA-PEG-PLGA was obtained at LA/GA from 1/1 to 3/1.^36^ Even though it was not significant of curvature (*P* > 0.05), the model did not meet the requirement to make it significant (*P* > 0.05). Particularly, all co-block polymers had no significant effect on the zeta potential (*P* > 0.05). Therefore, alteration of LA to GA molar ratio did not affect the surface charge of the NpM. However, all the co-block polymers were categorized as a physically stable formulation.

### The effect on entrapment efficiency and drug loading

**Figure 5:**
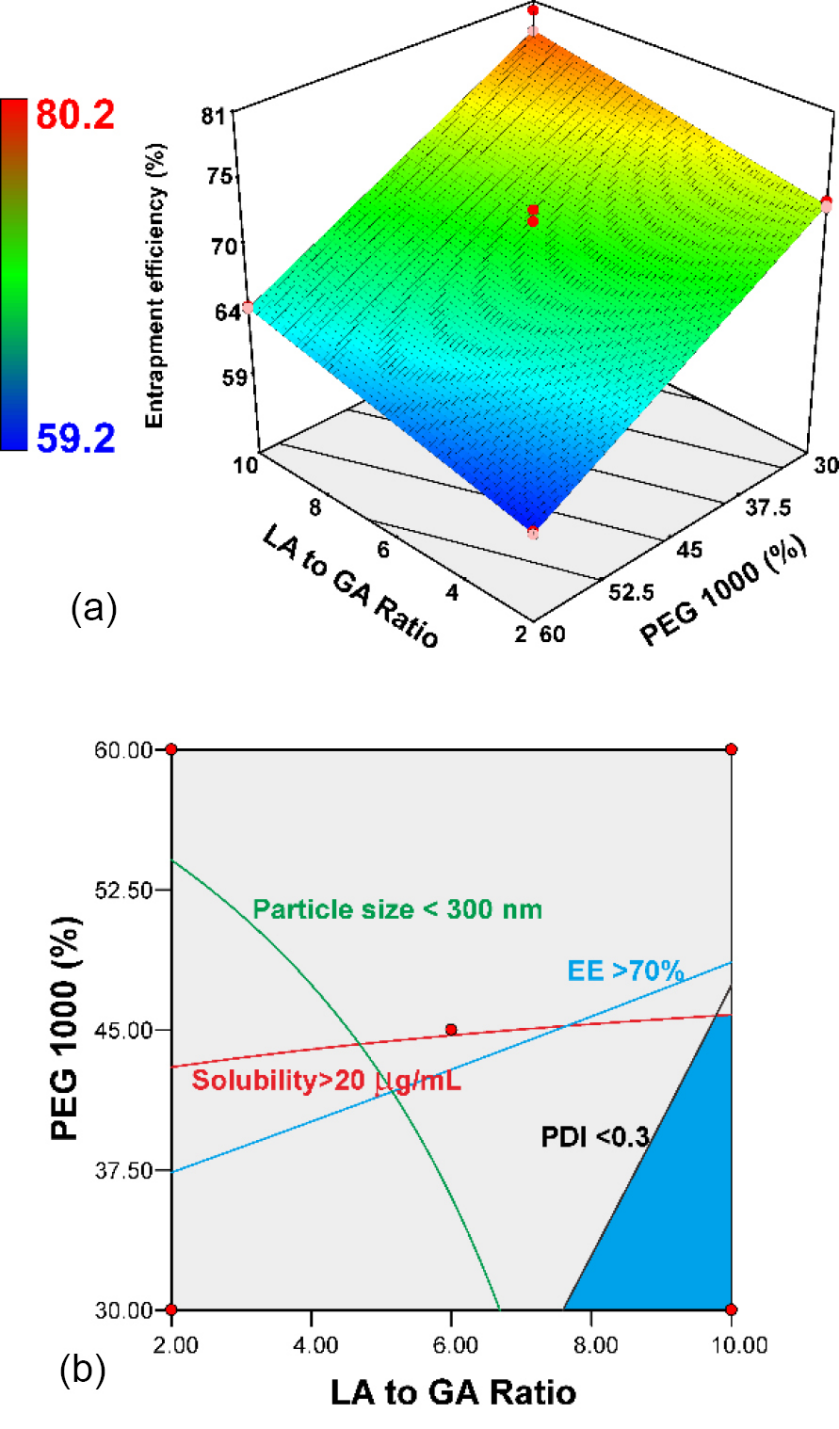
Contour plot of entrapment efficiency (a) and superimposed contour plot (b).

EE was correlated to solubility and particle size. In particular, the amount of entrapped lipophilic moiety had a linear function as there was an increase in solubility because of molecular entrapment and dynamic size. The EE for all co-blocks ranged from 59.2 to 80.2% and drug loading ranged from 2.37% to 3.21% wt. Both EE and drug loading were a similar response to describe the encapsulation effect. According to the MLRA approach, this design could obtain better EE results than reported by optimizing and adjusting the priority of the EE parameter.^36^ The EE model met all the requirements for prediction. The LA-to-GA ratio significantly increased EE while PEG reduced EE (*P* < 0.05). The PEG concentration (-6.99) had a more pronounced contribution effect than the LA-to-GA (+2.64) ratio. LA played fundamental roles in determining PLGA-PEG-PLGA co-block polymer owing to the first completely reacted with the other constituents and increasing hydrophobicity as well as hydrophobic interactions.^31,32^ The interaction between both factors was found to be significant (*P* < 0.05). The 3D-contour plot of EE is found in Figure 5a. The highest EE was obtained at high LA-to-GA ratios and low PEG concentrations. On the contrary, the lowest EE was obtained at the high PEG and a low LA-to-GA ratio. According to the contour plot, the unique interaction was not observed in the EE 3D-contour plot (Figure 5a). PLGA co-polymer was responsible to increase solubility and EE of SMV as well as modulation the particle size and distribution. In addition, the molecular weight of PLGA-PEG-PLGA contributed to an increase in EE indirectly through net sheer stress reduction owing to particle size enlargement.^36^

### Optimization of nano-polymeric micelle and NMR characterization for optimized co-block polymers

In order to determine the optimized co-block polymer, critical quality attributes depend on quality target co-block profiles as seen in Table 3. A superimposed contour plot was constructed depending on the contour plots of all responses (see Figure 5b). The optimized region of the co-block was limited by EE at more than 70%, particle size less than 300 nm, and solubility more than 20 µg/mL. Furthermore, the optimized region was obtained at LA-to-GA ratios from 9 to 10 and PEG concentrations of 30%-40%. The optimized co-block was obtained at an LA-to-GA ratio of 10% and 35.93% PEG. The optimized region was limited by the distribution of particle size profiles and solubility enhancement. The controlled space based on the superimposed contour plot was successfully verified, and there was an insignificant difference between the predicted and observed data (Table 3). In addition, the lower residual (<10%) was preferable. The observed results with regards to solubility and EE did not indicate a significant difference from the predicted results (*P* > 0.05) (Table 3). However, two parameters, i.e., particle size and distribution, did not adhere to what was expected, and the curvature was significantly different. This indicated that the curvature addition of the design model improved the predictive power. However, the contrary results passed the verification test owing to significant curvature. In this phase, the addition of a new point (middle level) to investigate particle size and distribution behavior was required. In other words, the optimized co-block polymer had been successfully verified statistically.

**Table 3:** Verification data of optimized nano-polymeric micelle delivery system

**Critical quality attributes**	**Predicted result**	**Observed result**	***P *** **value**	**Residual** ^b^ **(%)**
Solubility (µg/mL)^c^	30.3	31.0±2.19*	0.594	2.31
Particle size (nm)^d^	228.4	272.0±5.13	0.000	19.09
Polydispersity index^d^	0.2	0.3±0.02	0.007	50.00
Entrapment efficiency (%)^c^	75.8	69.9±0.2*	0.292	-7.78
Zeta potential (mV)	-19.38^a^	-18.5±0.37*	0.395	-4.54

^a^ bias result owing to not significant model ^b^ calculated based on the percentage of difference between predicted and observed results ^c^ the curvature found to be not significant (*P* > 0.05) ^d^ the curvature found to be significant (*P* < 0.05) * Statistically not significant difference (*P* > 0.05)

**Figure 6:**
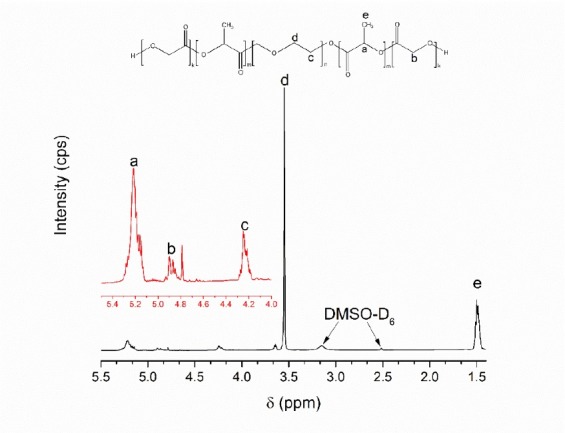
^1^H-NMR spectrum of optimized PLGA-PEG-PLGA co-block polymer. Overviewed spectrum was highlighted with red color. Notation of a, b, c, d and e was corresponding to the chemical shift of the proton in the functional groups of PLGA-PEG-PLGA

**Figure 7:**
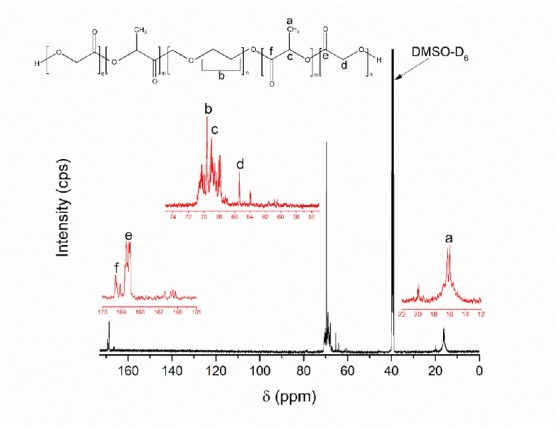
^13^C-NMR spectra of optimized PLGA-PEG-PLGA co-block polymer. Overviewed spectrum was highlighted with red color. Notation of a, b, c, d, e, and f was corresponding to the chemical shift of the carbon in the functional groups of PLGA-PEG-PLGA

The optimized co-block polymer had been fully characterized. The ^1^H NMR spectrum of the optimized PLGA-PEG-PLGA co-block polymer is presented in Figure 6. The specific peaks for chemical shifts according to the presence of protons in the PLGA-PEG-PLGA co-polymer were assigned at five peaks. Peaks at δ 1.50, 4.85, and 5.21 ppm were respectively assigned as protons of methyl, methylene, and methine groups in the PLGA co-polymer. Several studies confirmed that a chemical shift of 4.85 and 5.21 ppm corresponded to GA and LA, respectively.^28,36,37^ In addition, the methylene protons peak of PEG was assigned at δ 3.53 ppm. According to the Yu et al,^32^ the sequence of LA and GA was depicted by different patterns of peaks at 4.85 and 5.21 ppm. The optimized co-block followed an intermittent formation of LA and GA sequences and LA was more predominant than GA units.^32^ Meanwhile, the ^13^C-NMR spectrum is featured in Figure 7. Six specific peaks of carbon spectra of PLGA-PEG-PLGA co-block polymers were assigned. The carbon atoms of methyl, methylene, methine, and two carbonyls (from GA and LA monomers) in the PLGA co-polymer were assigned at δ 16.3, 65.4, 69.0, 168.7, and 169.3 ppm. The methylene group of the PEG co-polymer was assigned at δ around 68-70 ppm. The peak at δ 173.517 ppm was attributed to the carbonyl end group of the PLGA co-polymer. The NMR results suggested that the formation of PLGA-PEG-PLGA was confirmed by the end group of the PLGA co-polymer with specific peaks in the ^1^H and ^13^C NMR. Therefore, this proved the formation of PLGA on the side of the co-block polymers. This had also been confirmed by several reported studies.^21,32,38^

The molecular weight of PLGA-PEG-PLGA could be calculated based on Lu’s method.^38^ PEG 1000 has 88 mol of methylene proton. According to the methyl group of LA, the peak area ratio of methylene PEG and methyl LA was 1.28. Therefore, LA in the system equated to 22.9 mol. Thereafter, the methylene group of GA to methylene PEG peak area ratio was 25.6. As such, the GA in the co-block system was equivalent to 3.44 mol. Thus, the LA-to-GA stoichiometric ratio was 22.9/3.44 (6.65) and the average molecular weight of a PLGA-PEG-PLGA co-block polymer was 2848 Da ({22.9×72} + {3.44x58} + 1000). In other words, a higher ratio of LA to GA (22.4/1.6) promoted greater molecular weight.^32^ Another investigation noted that powder X-ray diffraction can be applied for this co-polymer’s characterization.^37^ However, we assumed that this method was not adequate owing to amorphous packing of the crystal structure of this polymer and semi-solid state.

## Conclusion

The formation of PLGA-PEG-PLGA was successfully confirmed by ATR-FTIR spectra, DSC thermogram, and NMR spectra. According to the DoE, LA-to-GA molar ratio had a higher effect on the particle size reduction. PEG contributed to reducing the solubility and entrapment efficiency of SMV. The simultaneous assessment showed that interaction both factors reduced the solubility of SMV and increased particle size and entrapment efficiency. Finally, LA-to-GA molar ratio and PEG concentration should be considered in the preparation of PLGA-PEG-PLGA co-block polymer affecting the formation and characteristics of NpM system.

## Ethical Issues

Not applicable.

## Conflict of Interest

Authors declare no conflict of interest in this study.

## Acknowledgments

The authors would like to thank Faculty of Pharmacy, Universitas Gadjah Mada for funding this research and Dexa Medica for providing the SMV. Syaiful Choiri would like to thank Indonesian Endowment Fund for Education (LPDP) for supporting this research.
